# Successful Management of Hydroxyurea-induced Leg Ulcers in Essential Thrombocythemia: Report of 3 Cases

**DOI:** 10.4274/Tjh.2012.0134

**Published:** 2013-06-05

**Authors:** Jihane Abou Rahal, Rim S. Ishak, Zaher K. Otrock, Joseph E. Maakaron, Samer Ghosn, Ali T. Taher

**Affiliations:** 1 American University of Beirut Medical Center, Department of Internal Medicine, Beirut, Lebanon; 2 American University of Beirut Medical Center, Department of Internal Medicine, Beirut, Lebanon

**Keywords:** Hydroxyurea, Essential thrombocythemia, Leg ulcers, Interferon

## Abstract

Essential thrombocythemia is one of the myeloproliferative neoplasms with a plethora of thrombohemorrhagic complications. Hydroxyurea has been proven to be an effective treatment for this condition. However, it is not without side effects. We herein report 3 patients with essential thrombocythemia treated with hydroxyurea who developed refractory leg ulcers, and we outline their successful management. We also review the literature to shed light on the mechanism of this toxicity. Awareness of this important treatment complication is important to avoid the pitfall of futile invasive interventions.

## Abstract (tr)

Esansiyel trombositoz, trombohemorajik komplikasyonların sık görüldüğü miyeloproliferatif hastalıklardan biridir.Hidroksiürenin bu durum için etkili bir tedavi olduğu kanıtlanmıştır. Bununla birlikte, yan etkisiz değildir. Bu makalederefrakter bacak ülserleri gelişen 3 esansiyel trombositoz hastasında hidroksiüre ile başarılı şekilde gerçekleştirilen tedavisürecini bildiriyoruz. Aynı zamanda, bu toksisitenin mekanizmasına ışık tutacak literatür gözden geçirilmiştir. Bu önemlikomplikasyonun farkında olmak, gereksiz invaziv girişimlerden kaçınmak için önemlidir.

## INTRODUCTION

Essential thrombocythemia (ET) is a BCR-ABL1-negative myeloproliferative disorder characterized by stem cell-derived clonal myeloproliferation leading to thrombocytosis. To establish a diagnosis of ET, reactive causes of thrombocytosis should be absent and other chronic myeloproliferative disorders should be ruled out [1]. An effective treatment for ET is hydroxyurea (HU), yet it is well known to cause several side effects, among them being HU-induced multiple and painful treatment-resistant leg ulcers [2]. We herein report 3 cases of HU-induced ulcers that were treated by discontinuation of HU and introduction of interferon, with marked improvement of the ulcers and good control of the ET.

## CASE 1

An 88-year-old Caucasian woman with a history of transient ischemic attack 17 years prior to presentation was referred to our clinic for anemia (hemoglobin= 11.0 g/dL; hematocrit= 32%), thrombocytosis (1100x10^9^/L), and splenomegaly. Her family history was positive for polycythemia vera and her bone marrow aspirate showed huge clumped platelets. A diagnosis of ET was made and she was started on HU and aspirin. She later tested positive for the JAK2 V617F mutation once the test became commercially available. She was started on HU (500 mg daily) and aspirin (100 mg daily) and responded well to treatment, with platelet count dropping to around (600x10^9^/L), correction of the anemia, and development of macrocytosis. She was also taking calcium, 5 mg of folic acid, and multivitamins. In 2004, platelet count started rising again and hemoglobin dropped. The HU dose was increased to 1000 mg twice a week and 500 mg for 5 days/week. She presented in 2011 with extensive cutaneous ulcerations of 2 months in duration over her right lateral malleolus. Upon diagnosis of HU-induced ulcer, the causative drug was discontinued and she was started on 90 µg of pegylated interferon alpha 2a (Pegasys®) to control her platelet count. Two weeks after discontinuing HU, the ulcerations drastically improved. Platelet count kept dropping and she is now maintained on 90 µg of Pegasys every 2–3 weeks.

## CASE 2

A 59-year-old Caucasian woman presented to our clinic after sustaining a myocardial infarction. Work-up revealed thrombocytosis (900x10^9^/L) . She also had splenomegaly on exam. A bone marrow aspirate showed hypercellular bone marrow with increased megakaryocytes, and mutational analysis showed the patient to be homozygous for the JAK2 V617F mutation. The diagnosis of ET was made and she was started on aspirin and HU; the dose was titrated to 1500 mg daily with an adequate erythroid and platelet response (~400x10^9^/L). In the fifth year of HU treatment, she developed skin ulceration over the plantar aspect of the soles bilaterally. At that time, she was taking low-dose aspirin and 1500 mg of HU 500. HU was then stopped and the patient was switched to anagrelide. However, she was not able to tolerate it due to dizziness and gastrointestinal distress. The patient was then shifted to 135 µg per week of Pegasys® with subsequent improvement in her platelet counts and healing of her ulcers. Platelet count is now adequately controlled and she only requires 90 µg of Pegasys every 2–3 weeks.

## CASE 3

A 76-year-old Caucasian woman presented in 2002 with a platelet count of (10x10^9^/L). Her spleen size was also on the upper limit of normal with a span of 13.1 cm. Mutational analysis for JAK2 V617F was negative. A bone marrow aspirate revealed significant clumping of platelets. She was started on HU and titrated to a dose of 1000 mg twice per week and 500 mg for the rest of the days. In 2009, she developed a gradual drop in hemoglobin and hematocrit (10 g/dL, 30%). Work-up revealed stage I adenocarcinoma of the colon, which was successfully resected. In 2010, she developed a left lower extremity ulcer measuring approximately 2x2 cm in size. She also developed dermatomyositis. At first, the ulcers were treated with surgical debridement and antibiotic administration. However, they did not heal despite the lack of bony involvement. The patient was diagnosed with HU-induced ulcers. Upon discontinuation of the drug, the ulcers started healing spontaneously and showed marked improvement within 3 months. Meanwhile, the platelet counts were rising. The patient was started on pegylated interferon alpha 2a (90-µg injections once weekly). The ulcers healed completely 6 months after HU was ceased, and the patient was continued on interferon with clinical and hematological remission up to 1 year after initiation of therapy. She is now adequately controlled with a Pegasys 90-µg injection once every 3 weeks.

## DISCUSSION

This report highlights an important and often undiagnosed toxicity associated with hydroxyurea and its successful management in ET patients. The benefit of HU treatment in ET is not a survival benefit since the patients already have a near normal survival rate, with 80% alive at 15 years [1]. Instead, the aim of treatment in ET is preventing thrombohemorrhagic complications, as well as dealing with the associated vasomotor disturbances (headache, lightheadedness, acral paresthesia, etc). Indeed, the risk of developing thrombosis reaches 20% in this population [1]. Paradoxically, in the case of platelet counts higher than 1000x10^9^/L, some patients may develop acquired von Willebrand syndrome [1]. Accordingly, ET patients are divided into either low risk or high risk for thrombosis. Table 1 shows the different risk strata for patients with ET along with their recommended treatment. Our patients were placed on HU since they had all suffered thrombotic events. For over 50 years, HU has been used in the treatment of chronic myeloproliferative disorders, such as chronic myeloid leukemia, polycythemia vera, and ET [3]. It is the hydroxylated derivative of urea, which works by blocking the ribonucleotide-diphosphate reductase, thus inhibiting the synthesis of DNA and ultimately leading to cell death in the S phase of the cell cycle. Its cytotoxic effects are most prominent in the bone marrow and in epithelial cells. HU is usually well tolerated with few side effects [4]. Among its adverse effects are bone marrow depression, megaloblastosis, fatigue, headache, fever, and gastrointestinal symptoms [4]. The drug has also been reported to cause an array of dermatological reactions that include alopecia, skin or fungal hyperpigmentation, poikiloderma, erythematous scaling eruptions, atrophy of the skin and subcutaneous tissues, erythema and scaling of acral sites simulating chronic dermatomyositis, lichen planus-like lesions, and skin tumors on UV-exposed areas [4,5,6,7,8,9]. In addition, leg and oral ulcers have been documented in patients taking HU [9].

**Figure 1:**
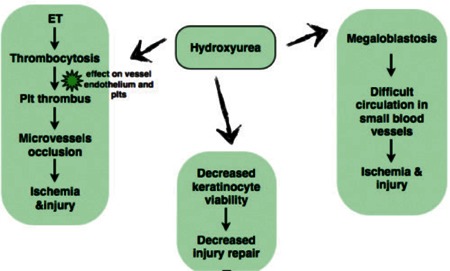
Possible mechanisms leading to hydroxyurea-induced ulcers.

The leg ulcers caused by HU typically occur on the lateral malleolar area and have been reported in approximately 9% of patients taking hhigher doses of HU for a period exceeding 1 year [3,10]. These extremely painful ulcers, although small and superficial, rarely heal if the medication is not withdrawn [3]. Although the exact mechanism by which HU causes these ulcers is unclear, 3 possible mechanisms explain how HU causes cutaneous ulcerations in a patient with ET (Figure 1). First, the thrombocytosis that is associated with ET, coupled to a poorly understood effect of HU on vessel endothelium and platelets, is likely to result in platelet thrombus with subsequent transient occlusion of microvessels [3]. Megaloblastosis (a known side effect of HU) has been postulated to play a role, too, as the enlarged red blood cells circulate less easily in small blood vessels [3,10]. Because it promotes cell death, HU can decrease keratinocyte viability, which in turn will hinder any re-epithelialization after the injury done by the above mechanisms. The effect is typically most visible on surfaces prone to mechanical trauma, such as the malleolar areas [3,10]. 

The cornerstone of treatment of HU-associated ulcers is discontinuation of HU. Indeed, the ulcerations rarely respond to conventional optimal therapy [2,3,5,8,10,11]. They appear to be even refractory to surgical treatment if HU is not discontinued, as evidenced by a failed flap in the case reported by Tsuchiya et al. [3] in 2010 as well as in our own experience with the third case. Being a rarely encountered side effect of HU, HU-induced ulcers are frequently underdiagnosed, thus delaying their appropriate management. Even when appropriately diagnosed, the physician is often faced with the inability to discontinue HU in order to keep the original disease under control [11]. This leads to a delay and/or inability in healing the ulcers, putting the patient at risk of functional and psychological discomfort as well as potential superimposed infections. In addition, the patients are typically resumed on HU after healing of the wounds, despite a high risk for the ulcerations to recur upon reintroducing the HU [11]. Our 3 patients with HU-induced ulcers healed completely after discontinuation of HU. When they were shifted to interferon therapy (Pegasys®), it maintained good control over their primary disease, comparable to that of HU. This degree of disease control with interferon is in accordance with findings of previous studies, which established that interferon-alpha 2a adequately controls thrombocytosis and vasomotor symptoms in ET [12].

As a conclusion this report demonstrates the complication of hydroxyurea-induced leg ulcerations and outlines successful management options. Our experience shows complete regression of the ulcers and adequate control over the primary disease after discontinuation of HU and replacement with an effective agent. Alternative treatment with interferon seems to be a viable option, especially given the risk of recurrence of ulcers when HU is resumed. It also offers a treatment substitute in cases where discontinuation of HU without replacement is not possible due to the need of tight control over ET.

## CONFLICT OF INTEREST

ATT serves on the Novartis Speakers Bureau. He also receives research funding from Novartis. The other authors declare that they have no conflict of interest. This study did not receive any external funding. 

## Figures and Tables

**Table 1:**
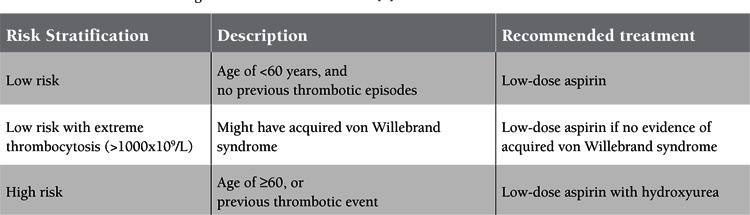
Classification of ET along with recommended treatment [1].
